# Comparing effectiveness of physiotherapy versus drug management on fatigue, physical functioning, and episodic disability for myalgic encephalomyelitis in post-COVID-19 condition: a study protocol of randomized control trial

**DOI:** 10.1186/s13063-024-08077-x

**Published:** 2024-05-15

**Authors:** Altaf Hossain Sarker, K.M. Amran Hossain, Md. Feroz Kabir, Sharmila Jahan, Md. Zahid Hossain, Tofajjal Hossain, Iqbal Kabir Jahid

**Affiliations:** 1 Department of Microbiology, Jashore University of Science and Technology (JUST), Jashore, 7408 Bangladesh; 2 Department of Physiotherapy and Rehabilitation, Jashore University of Science and Technology (JUST), Jashore, 7408 Bangladesh

**Keywords:** Myalgic encephalomyelitis, Post-COVID-19 condition, Physiotherapy, Drug, Randomized controlled trial

## Abstract

**Background:**

Physiotherapy interventions effectively improved fatigue and physical functioning in non-COVID patients with myalgic encephalomyelitis or chronic fatigue syndrome (ME/CFS). There is a research gap on the effectiveness of physiotherapy interventions versus drug management on ME/CFS in post-COVID-19 conditions (PCC).

**Methods:**

We planned a three-arm prospective randomized control trial on 135 PCC cases with ME/CFS who are diagnosed between 20 November 2023 and 20 May 2024 from a population-based cohort. The study aims to determine the effectiveness of physiotherapy interventions as adapted physical activity and therapeutic exercise (APTE) provided in institution-based care versus telemedicine compared with drug management (DM). Participants will be assigned to three groups with the concealed location process and block randomization with an enrollment ratio of 1:1:1. The post-treatment evaluation will be employed after 2 months of interventions, and follow-up will be taken after 6 months post-intervention. The Chalder fatigue scale will measure the primary outcome of fatigue. SF-36 and the disability-adjusted life years (DALYs) will measure the secondary outcome of physical functioning and episodic disability.

**Discussion:**

This study will address the research gap to determine the appropriate approach of physiotherapy or drug management for ME/CFS in PCC cases. The future direction of the study will contribute to developing evidence-based practice in post-COVID-19 condition rehabilitation.

**Trial registration:**

The trial is registered prospectively from a primary Clinical Trial Registry side of WHO CTRI/2024/01/061987. Registered on 29 January 2024.

**Supplementary Information:**

The online version contains supplementary material available at 10.1186/s13063-024-08077-x.

## Introduction

Post-COVID-19 condition (PCC) can be defined as persistent symptoms for more than 12 weeks of SARS-COV-2 diagnosis that lasts for at least 2 months and is not related to any other clinical diagnosis [1]. This clinical case definition of PCC by WHO working group is more specific in terms of diagnosis and explaining the symptom responses causing episodic disability [2]. The prevalence of post-COVID-19 condition is globally estimated at 43%, and in Asia 51%, of all COVID-19 cases [3]. Two Bangladeshi large-scale studies found a different prevalence of PCC also known as long COVID between 16.1% [4] and 24% [2, 5]. The symptom responses of PCC are diverse; among all the symptoms, pain and fatigue are prominent [2–6].

Myalgic encephalomyelitis or chronic fatigue syndrome (ME/CFS) is an ongoing multisystem relapsing–remitting symptom characterized as fatigue associated with pain [7] that can be evident in any post-viral sequelae. ME/CFS impacts the cognitive, immune, and autonomic nervous systems [7, 8]. This is a disabling condition with a poor progression that significantly impacts the activity and performance of an individual [9]. One study finds that ME/CFS is one of the prevalent conditions among PCC cases, with a global prevalence nearing 45% of all post-COVID-19 (PCC) cases [10]. In the USA, 2.5 million people suffer from ME/CFS for different reasons [11]. As per the disease burden, PCC has less research and funding [12]. ME/CFS affects a person’s daily activities and participation in livelihood, including physical and psychological state [10]. Moreover, PCC is clinically related to impaired physical functioning and reduced quality of life [13]. ME/CFS have a wide range of symptoms; the key clinical features include fatigue and pain; the additional symptoms are headache, photophobia, problems in short-term memory, reduced ability to multitask works, brain fog, and difficulty in working online or even watching television [14].

The management of ME/CFS is symptomatic, which includes managing underlying pain symptoms, fatigue, brain fog, sleep issues, musculoskeletal problems, and postural tachycardia syndrome (POTS) [15, 16]. ME/CFS symptoms are treated with a variety of medications such as amphetamine, methylphenidate, naltrexone, duloxetine, gabapentin, intravenous solution, Dexedrine, fludrocortisone, trazodone, clonazepam, tricyclic antidepressants, ketotifen, montelukast, diphenhydramine, and metoprolol [15, 16]. Other management by medications for ME/CFS includes treating with azithromycin, remdesivir, favipiravir, infliximab, tocilizumab, siltuximab, hydrocortisone, rituximab, rintatolimod, and intravenous immunoglobulin [17, 18]. The overall physiotherapy management for ME/CFS aims to improve the painful status, cardiorespiratory functions, adaptive coping for fatigue, energy consumption and restoration, and improving physical and psychological well-being [19]. Physiotherapy management of ME/CFS cases includes exercise, pacing, and different indicative approaches such as cognitive behavioral therapy [19, 20]. A wide range of exercise therapies can be prescribed for ME/CFS, including customized exercise, aerobic exercise, and adapted physical activity and therapeutic exercise programs [11, 19, 20]. Evidence suggests adaptive physical activity and therapeutic exercise programs are more effective than passive control or cognitive behavioral therapy for non-COVID patients having ME/CFS [13].

The physiotherapy management for ME/CFS must adhere to safe post-COVID-19 condition rehabilitation [21] and NICE guidelines [22]. A study examined the effect of exercise on patients with ME/CFS in non-COVID patients and found promising results in favor of exercise therapy [19]. The key outcome of exercise in ME/CFS is restoration of physical functioning that is significantly improved even after 12 to 24 weeks of interventions [22–25]. In recent studies, the adapted physical activity and therapeutic exercise programs (APTE) have been considered superior to other exercise programs, patients’ education, or active control for non-COVID cases having ME/CFS [26]. As there is no study exploring the outcome of APTE on ME/CFS in PCC cases, our hypothesis is that APTE can be more effective in reducing fatigue and improving physical functioning than drug management for PCC cases having ME/CFS. Our study aims to determine the effectiveness of adapted physical activity and therapeutic exercise programs (APTE) through institution-based care (APTE-I) versus telemedicine (APTE-T) compared with drug management (DM) on fatigue, physical functioning, and episodic disability for PCC cases having ME/CFS. The objectives are to (1) find baseline compatibility among APTE-I, APTE-T, and DM; (2) determine the among-group, among-observation outcomes on fatigue, physical function, and episodic disability for PCC patients having ME/CFS; and (3) present the post hoc within-group, within-observation outcomes of APTE-I, APTE-T, and DM.

## Methodology

### Study design

The proposed study will be a three-arm randomized clinical trial (RCT) of PCC patients diagnosed with ME/CFS according to WHO working group criteria [1]. PCC cases having ME/CFS will be randomized to three separate groups: adapted physical activity and therapeutic exercise provided in an institution-based setting (APTE-I), adapted physical activity and therapeutic exercise provided through telemedicine (APTE-T), and drug management (DM) group. Participants will be enrolled from a population-based inception cohort of post-COVID-19 cases [2, 6], with defined eligibility criteria.

### Sample size calculation

The sample size was calculated using the software ClinCalc [27], and the key primary outcome was determined as the score of fatigue in the Chalder fatigue scale (CFS) [28]. Sample size has been calculated as the minimal clinically important differences (MCID) of CFS were estimated at 9.14 ± 2.73 in 0–33 Chalder fatigue scale, and superiority is considered as a 15% improvement from the baseline. The enrolment ratio will be 1:1:1. The calculation was completed with 80% power and an alpha value of 0.05. With the calculation, the total sample stands at 124, and each group will have a minimum sample of 42. For safety purposes, we considered each group sample to be 45.

$$k=\frac{n1}{n2}=1$$

$${n}_{1}=\frac{\left({\sigma }_{1}^{2}+\frac{{\sigma }_{2}^{2}}{K}\right){\left({z}_{1-\frac{\alpha }{2}}+{z}_{1-\beta }\right)}^{2}}{{\Delta }^{2}}$$

$${n}_{2}=K\times {n}_{1}$$

Δ =|${\mu }_{2}-{\mu }_{1}$|= absolute difference between two means,

${\sigma }^{1},{\sigma }^{2}$ = variance of means #1 and #2

${n}_{1}$ = sample size for group #1 ${n}_{2}$= sample size for group #2

*α* = probability of type I error (usually 0.05)

*β* = probability of type II error (usually 0.2)

*z* = critical *Z* value for a given *α* or *β*

*k* = ratio of sample size for group #2 to group #1

### Study duration

Participants will be recruited between 21 May 2024 and 20 August 2024. Baseline compatibility will be conducted on the initial recruitment day, followed by the intervention and outcome evaluation. The post-treatment assessment will be performed after 2 months of the initial recruitment, and the follow-up will be undertaken after 6 months of post-intervention evaluations.

### Study population, samples, and eligibility criteria

The post-COVID-19 condition (PCC) cases of ME/CFS will be screened from the inception cohort [6] of post-COVID cases. The respondents from Dhaka and Khulna divisions will be considered a study population. The eligibility criteria for inclusion in the study will be participants (1) aged 18 years or above, (2) COVID-19 symptoms onset at least 12 weeks and perseverance at a minimum of 8 weeks [1], (3) diagnosed PCC according to WHO working group criteria [1], (4) diagnosing ME/CFS according to 2006 Canadian consensus criteria [29], (5) willing to participate in the trial with consent of adherence with the interventions, and (6) eligible for drug management according to the physician’s assessment. Exclusion criteria will be (1) any preexisting post-exertion symptom exaggeration, (2) any preexisting clinical condition with fatigue such as cardiovascular or neurological disability, (3) any red flags or signs that are explained as contraindication according to safe post-COVID-19 condition rehabilitation guideline [30], and (4) patient drop-out within the 1st week of inclusion.

### Study settings

ME/CFS patients will be recruited and treated in three specialized hospitals. The study will be conducted in BRB Hospital Limited and Specialized Physiotherapy Hospital Ltd. in the Dhaka division. In the Khulna division, the treatment center will be the Department of Physiotherapy and Rehabilitation at Jashore University of Science and Technology. The APTE-I, APTE-T, and DM groups will be recruited from any centers. To prevent cross-contamination of the data, different treatment set-ups will be arranged in each study setting, and separate personnel will be employed for each treatment group.

### Study procedure

We will adopt a randomized sample enrollment and recruitment process in the trial. The respondents will be recruited from the inception cohort through the hospital-based randomization process. In group allocation, concealed allocation will be employed for APTE-I, APTE-T, and DM groups, and block randomization will be adopted. We will follow the standard criteria for maintaining the protocols as per Standard Protocol Items: Interventional Trials 2013 (SPIRIT guidelines) to ensure the rigor of designing the trial protocol (Table 1).

**Table 1:**
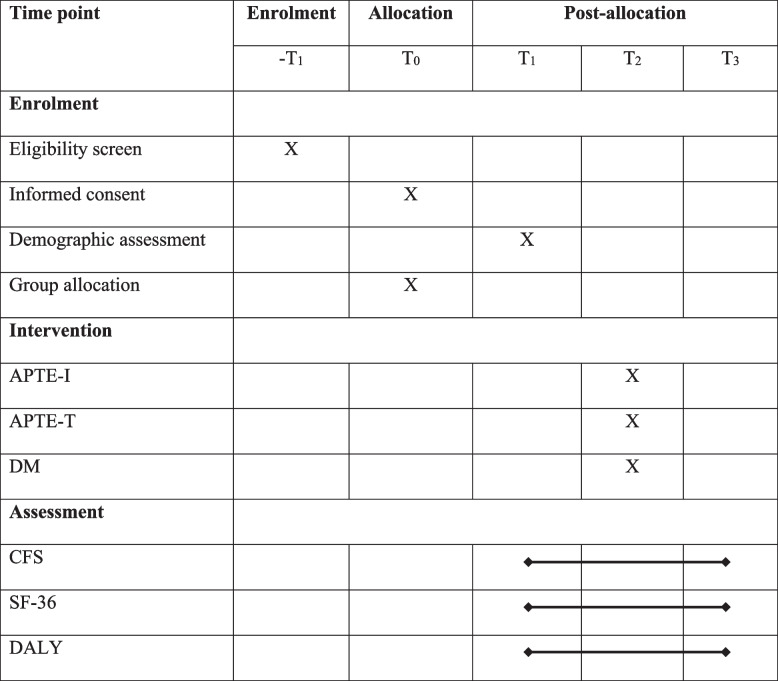
SPIRIT protocol for the study

*APTE-I* institution-based adapted physical activity and therapeutic exercise program, *APTE-T* telerehabilitation-based adapted physical activity and therapeutic exercise program, *DM* drug management, *CFS* Chalder fatigue scale, *SF-36* Short Form 36 Health Survey Questionnaire, *DALY* disability-adjusted life years, *T*_*0*_ group allocation, *T*_*1*_ baseline before the intervention, *T*_*2*_ measurement taken in 2 months after *T*_1_, *T*_*3*_ measurement taken after 6 months of *T*_2_

### Recruitment strategies

The population of the study will be the diagnosed ME/CFS cases of inception cohort conducted in Bangladesh. We will have a pool of selected samples who have the disease and are aged more than 18 years, and we will do simple random sampling through random numbers to allocate the groups and centers by Excel “rand” function. The allocation will be computer-generated and concealed.

### Interventions

The intervention protocol for APTE-I and APTE-T will be according to an e-Delphi consensus [31], systematic review [32], Cochrane review [13], clinical trial [26], and a systematic review research from a research group in Norway [19]. The APTE protocol will consist of breathing exercises and breathing control exercises [32], exercises to improve flexibility and motor activities [13], aerobic capacity exercise [13, 19, 32], and interventions to maintain a healthy lifestyle [19, 31, 32]. All the interventions will be provided as per the recommendation from Safe Post-COVID-19 condition rehabilitation [30]. To ensure a safe application of interventions, we will screen the participants for red flag signs and then prescribe personalized interventions supervised by a registered physiotherapist. In each session, patient feedback will be taken to ensure nothing is causing harm to the patient.

#### Institute-based adapted physical activity and therapeutic exercise (APTE-I)

APTE-I will be provided under the consultation of a consultant physiotherapist specializing in post-COVID-19 condition rehabilitation. Interventions will be provided in 45-min sessions in a one-to-one approach. There might be some home exercises or advice to follow at home. There will be continuous communication with patients, to ensure that the treatment does not deteriorate the symptoms.

#### Adapted physical activity and therapeutic exercise through telemedicine (APTE-T)

For APTE-T, the interventions will be provided by a consultant physiotherapist through digital media. such as Zoom, WhatsApp, Facebook Messenger, or the personalized app of the Department of Physiotherapy and Rehabilitation at Jashore University of Science and Technology. It will be a 45-min session with a one-to-one approach. The patient will be performing exercises or advice at home. The physiotherapist will explain and demonstrate procedures through cameras, and the patient will perform. There will be continuous communication with the patients to ensure that the interventions provided are performed accordingly and are safe.

#### Drug management (DM)

Participants of the drug management group will receive drug interventions as azithromycin, remdesivir, favipiravir, infliximab, tocilizumab, siltuximab, hydrocortisone, rituximab, rintatolimod, and intravenous immunoglobulin [17, 18]. The drug interventions will be directly prescribed by a physician specialized in treating PCC cases. A single brand name will be prescribed for each drug. We will communicate with the patients to ensure no adverse effects of the medications. The patient will be given a choice if they are willing to join the exercise programs; they have full liberty to join the programs even after the completion of the trial.

### Treatment progression

The participants of all three groups will be performing exercise or taking their treatment for 8 weeks. The exercise group will take the interventions formally twice a week for 8 weeks with continuous monitoring. In case of any adverse effect, additional sessions will be employed depending on the opinion of the consultant physiotherapist or physician. The overall treatment for ME/CFS will be provided actively for 2 months, and after that, the treatment will stop, and there will be a 6-month follow-up.

### Outcome measures

#### Chalder fatigue scale (CFS)

Fatigue is the primary outcome of the study, and Chalder fatigue scale (CFS) [28, 33] will be used to measure the outcome of fatigue. CFS is an 11-item questionnaire with each item corresponding to a 4-point Likert scale [33]. A higher score indicates a higher level of fatigue. CFS is a valid and reliable scale with an agreeable internal consistency and a coefficient of 0.78–0.96 [34].

#### Physical functioning sub-domain of SF-36

The secondary outcome will be physical functioning. It will be determined through the physical function measures of SF-36 [35–37]. SF-36 is a valid and reliable tool for measuring health status with eight subdomains, one of which is physical functioning items [38]. Scores in the physical functioning section range from 0 to 100 [39, 40]. The larger scale reflects that the participants can perform all physical activities, and the lower scale indicates a limitation in physical function [41]. SF-36 has a reliability score of 0.80, and the physical functioning section reliability score is 0.90 [39].

#### Disability-adjusted life years (DALYs)

Another secondary outcome will be episodic disability of PCC, which will be measured by calculating disability-adjusted life years (DALYs). DALYs will be calculated by the sum of years of healthy life lost due to disability (YLDs) and years lost due to premature mortality (YLLs). For YLDs, the incidence of ME/CFS from the previous study (I), disability weight of PCC (DW), and duration of ME/CFS in weeks (*L*_1_) will be determined. The YLLs will be calculated by estimating the number of deaths due to COVID-19 in Bangladesh (N) and estimating *L*_2_ by deducting the mean age of death people from COVID-19 from the average life expectancy in Bangladesh.

The original questionnaire was formulated in English. Then, a bilingual researcher who is not involved in this study project translates forward Bangla to backward English.

### Study guideline

**Fig. 1:**
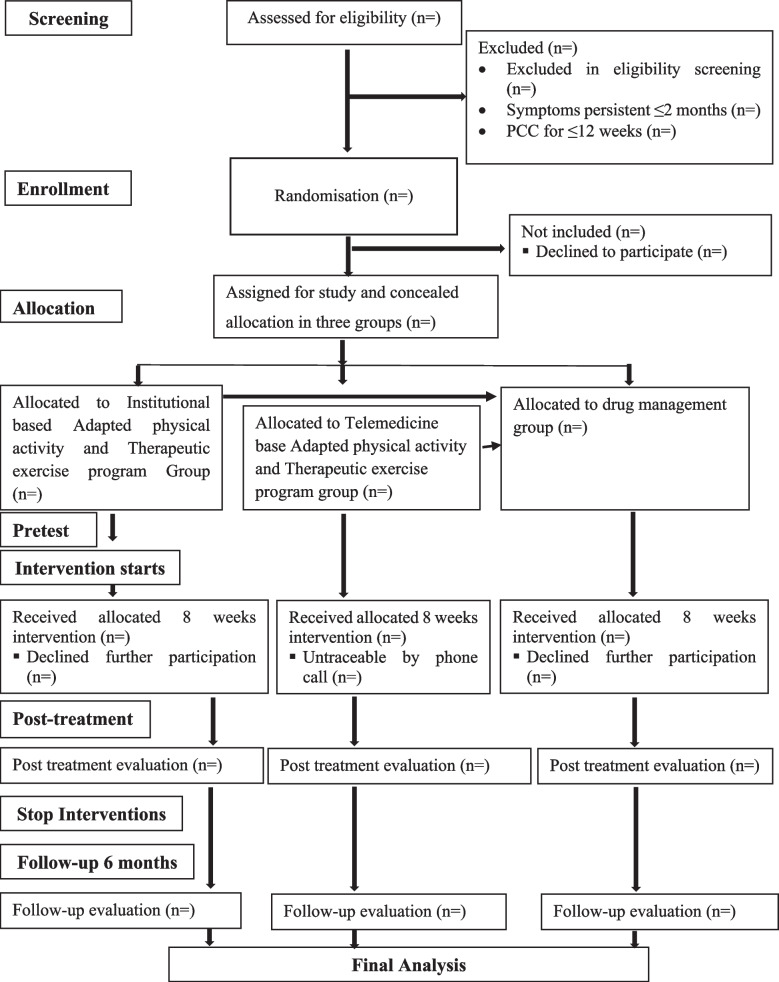
CONSORT flow diagram of the proposed trial

The study will follow the Consolidated Standards of Reporting Trials (CONSORT) guidelines mentioned in Fig. 1.

### Minimization of bias and blinding process

Three separate independent blinded assessors will document the baseline posttest and follow-up. All the intervention teams will be separate. The respondents of the physiotherapy groups will be blinded to the group allocations along with the treatment providers. The treatment providers will be aware of the treatment and treatment components, but they will be blinded to the group allocations. Participants will not pay for any interventions or be given any remunerations. The overall research team will not participate in any intervention or assessment. Training or demonstrations will be provided for data collectors, but no other measures will be taken.

### Monitoring data quality

There will be a separate data management team and one trial manager to ensure the rigor of the entire trial procedure. Only auditing and investigating the outliers will be done, and there will be no manipulation. There will be one internal and one external data monitoring personnel to ensure good clinical practice.

### Clinical monitoring team

A monitoring team consisting of two physiotherapists will monitor the trial procedure, especially monitoring the participants and ensuring that they are following the treatments. They will also ensure that there are no symptoms of exacerbation after the treatment. In case of any irregularities, they will report directly to the trial manager.

### Safety measures and managing adverse effects

We anticipate no adverse effect for the physiotherapy groups except minor post-exertional symptom exacerbation. We will follow safe post-COVID-19 condition rehabilitation guidelines [30] and maintain subjective and objective analysis and plan (SOAP note) to ensure safe interventions. For drug management, the participants will be monitored closely, and in case of any adverse effects, the responsible physicians will be informed. All the adverse effects observed will be monitored, documented, and recorded during the final publication of the study.

### Participate

Screening participants will be informed about the study’s aims, objectives, and intervention process and provided a written informed consent form. For online patients, informed consent will be obtained using a Google form and a verbal procedure. The full trial has ethical approval from the Institutional Review Board on the Institute of Physiotherapy, Rehabilitation & Research (IPRR) BPA-IPRR/IRB/19/01/2023/69 [13/02/2023] as part of a PhD project. The trial is registered to the Clinical Trial Registry India (CTRI), the primary trial site of the World Health Organization CTRI/2024/01/061987 [registered on: 29 January 2024]. We will follow the Helsinki Declarations’ ethical guidelines per the rules provided by the ethical approval bodies. Before enrolling, we will provide written informed consent and ensure the participation is voluntary, and they can withdraw the trial anytime during the trial. Also, the participants will be ensured that withdrawal from our study will not change their treatment process. The trial manager, principal investigator, and data auditors will have access to the final trial data set. After completing the trials, all the authors will have equal access to the anonymous data. All the hard copies and soft copies of data collection will be kept to the principal investigator, and there will not be any disclosure or access to the identification of trial patients. There will be post-trial care only if any adverse effects are noted during the trial.

### Data analysis

Data will be analyzed through Statistical Packages of Social Science (SPSS) version 23 for Windows. Normality will be examined through the Kolmogorov-Simonov and Shapiro–Wilk tests [42]. The descriptive analysis will be completed using the mean and the standard deviation for continuous variables and frequency and percentage for categorical variables. A baseline compatibility test will be performed using one-way ANOVA or Friedman’s ANOVA according to the data distribution. The within-group changes among three measurements or groups will be performed using one-way ANOVA or Friedman’s ANOVA and subsequent post hoc tests [43]. The among-group, among-observation changes will be determined using MANOVA or the multivariate Kruskal–Wallis test. We will use the intention-to-treat analysis. The significance level will be set as alpha value < 0.05, and subsequent Bonferroni correction will be made for post hoc tests. No interim analysis will be done.

### Trial status

Trial version number is 1, and the protocol date was 3 August 2023. This study will be recruiting participants from 21 May 2024 to 20 August 2024.

### Dissemination

After the completion of the trial, the result will be presented in the seminars to the relevant stakeholders in Bangladesh. Final research records will be presented and submitted to an indexed journal for publication. There will be a dissemination session for the physiotherapist and physicians to manage ME/CFS in post-COVID-19 conditions. In accordance with the criteria set forth by the International Committee of Medical Journal Editors (ICMJE), authorship for publications regarding trial results will be determined. We have a plan to publish the unanimous data.

## Discussion

ME/CFS has an overall disease burden double of HIV and half of breast cancer in the USA [44]. From a global perspective, it is estimated that nearly 45% of the global PCC survivors have ME/CFS [13]. The study is designed for a significant study population. According to previous studies, COVID-19-related low back pain [45], ME/CFS-associated pain, fatigue, and impaired physical functioning impact overall activity limitation, participation restriction, and environmental and social factors proceeding towards disabilities [19, 30–32]. There is limited evidence on pharmacological management [24], and a few nonpharmacological trials have been examined on the ME/CFS in non-COVID cases [13, 17, 20, 23, 24]. The proposed study will cover a significant research gap in estimating the outcome of physiotherapy compared with drug management for PCC cases having ME/CFS. In this study, we are ensuring safe treatment approaches according to existing guidelines [17, 18, 30]. The interventions also adhere to the Bangladeshi setup [31, 32]. Also, the study procedure adhered to standard protocols such as SPIRIT and CONSORT. Different layers of blinding and masking will be adopted to prevent the cross-contamination of the data. We have chosen valid and reliable tools for measuring the primary and secondary outcomes. The results of the studies will be confined to specific samples derived from 2 divisions of Bangladesh. However, the samples will be framed through a randomized process so that the findings will outweigh the limitations.

## Implication of the study

The study’s implication will contribute to a paradigm shift in treatment approaches for ME/CFS. The trial’s key strength is covering the recommended research gap, and this study will be implicated in the clinical management of ME/CFS for PCC cases. The study will enrich the body of knowledge of intervention protocols in post-COVID-19 condition rehabilitation or any post-viral sequel rehabilitation globally.

In conclusion, the overall study process is well-designed, synchronized, and well-planned to be executed through a complicated sequential process. We expect the findings to guide good clinical practice for managing ME/CFS for PCC cases.

### Supplementary Information

**Supplementary Material 1. **

**Supplementary Material 2. **

**Supplementary Material 3. **

**Supplementary Material 4. **

## Data Availability

There is no available data as this is a trial protocol.

## Abbreviations

CFS: Chronic fatigue syndrome
CBT: Cognitive behavioral therapy
APTE: Adapted physical activity and therapeutic exercise program
AC: Active control
MCID: Minimal clinically important differences
DALYs: Disability-adjusted life years
