# Supplementary material for: Comparing effectiveness of physiotherapy versus drug management on fatigue, physical functioning, and episodic disability for myalgic encephalomyelitis in post-COVID-19 condition: a study protocol of randomized control trial
Source: Trials. 2024 May 15;25:321. doi: 10.1186/s13063-024-08077-x (PMC11094988; doi:10.1186/s13063-024-08077-x)
Supplement: Supplementary file 1 — Supplementary Material 1. [file 13063_2024_8077_MOESM1_ESM.pdf]

FULL DETAILS (Read-only) -> Click Here to Create PDF for Current Dataset of Trial

**CTRI No**	**CTRI/2024/01/061987** [Registered on: 29/01/2024] **Trial Registered Prospectively**
**Acknowledgement Number**	REF/2024/01/077243
**Last Modified On:**	11/01/2024
**Post Graduate Thesis**	Yes
**Type of Trial**	Interventional
**Type of Study**	Drug<br>Physiotherapy (Not Including YOGA)
**Study Design**	Randomized, Parallel Group, Multiple Arm Trial
**Public Title of Study**	Physiotherapy for Post-COVID-19 Conditions
**Scientific Title of Study**	Comparing Effectiveness of Physiotherapy versus Drug Management on Fatigue, Physical Functioning, and Episodic Disability for Myalgic Encephalomyelitis in Post-COVID-19 Condition
**Trial Acronym**	PCCPT

Secondary IDs if Any

Secondary ID	Identifier
NIL	NIL

Details of Principal Investigator or overall Trial Coordinator (multi-center study)
Clarification(s) with Reply
Modification(s)

**Name**	Altaf Hossain Sarker
**Designation**	Professor
**Affliation**	Jashore University of Science and Technology (JUST)
**Address**	Room No 302, Department of Physiotherapy and Rehabilitation, M R Khan Medical Center, Jashore University of Science and Technology, Jashore-7408, Bangladesh<br><br>7408<br>Other
**Phone**	8801571297407
**Fax**	
**Email**	altafhossainsarker272@gmail.com

Details Contact Person Scientific Query

**Name**	Dr Iqbal Kabir Jahid
**Designation**	Professor
**Affliation**	Jashore University of Science and Technology
**Address**	Department of Microbiology, Jashore University of Science and Technology (JUST) Jashore Bangladesh<br><br>7408<br>Other
**Phone**	8801709818178
**Fax**	
**Email**	ikjahid_mb@just.edu.bd

Details Contact Person Public Query
Clarification(s) with Reply
Modification(s)

**Name**	K M Amran Hossain
**Designation**	Lecturer
**Affliation**	Jashore University of Science and Technology
**Address**	Room No 302, Department of Physiotherapy and Rehabilitation, M R Khan Medical Center, Jashore University of Science and Technology, Jashore-7408, Bangladesh

	7408<br>Other
**Phone**	01778790798
**Fax**	
**Email**	kma.hossain@just.edu.bd

**Source of Monetary or Material Support**	Physiotherapy unit, BRB Hospitals Ltd, Panthapath, Dhaka-1205, Bangladesh

Primary Sponsor

**Name**	Jashore University of Science and Technology
**Address**	Jashore University of Science and Technology Jashore-7408 Bangladesh
**Type of Sponsor**	Government funding agency

Details of Secondary Sponsor

Name	Address
NIL	NIL

Countries of Recruitment

Bangladesh

Sites of Study Clarification(s) with Reply Modification(s)

No of Sites = **1**

Name of Principal Investigator	Name of Site	Site Address	Phone/Fax/Email
Dr Tofajjal Hossain	BRB Hospital Limited and Specialized Physiotherapy Hospital Ltd	Room No 202, Department of Physiotherapy, BRB Hospital Limited,77A Panthapath, Dhaka 1215, Bangladesh	01715560669<br>yousufraju6423@gmail.com

Details of Ethics Committee

No of Ethics Committees= **1**

Name of Committee	Ethics Committee registered with DHR /CDSCO or not	Ethics Committee Registration No.	Approval Status	Date of Approval	Approval Document	Is IEC?
Institute of Physiotherapy, Rehabilitation & Research	Yes	BPA-IPRR/IRB/19/01/2023/69	Approved	13/02/2023	Approval File	Not Available

Regulatory Clearance Status from DCGI

Status	Date	Aproval Document
Not Applicable	No Date Specified	No File Uploaded

Health Condition / Problems Studied

Health Type	Condition
Patients	**(1) ICD-10 Condition:** J984||Other disorders of lung,

Intervention / Comparator Agent Clarification(s) with Reply Modification(s)

Type	Name	Details
Intervention	Adapted physical activity and therapeutic exercise through telemedicine (APTE-T)	3 Months. APTE-T the interventions will be provided by a consultant physiotherapist through digital media Such as Zoom, WhatsApp, Facebook Messenger, or the personalized app of the Department of Physiotherapy and Rehabilitation at Jashore University of Science and Technology. It will be a 45-minute session with a one-to-one approach. The patient will be performing exercises or advice at home. The physiotherapist will explain and demonstrate procedures through cameras, and the patient will perform. There will be

		continuous communication with the patients to ensure that the interventions provided are performed accordingly and is safe.
Comparator Agent	Drug Management (DM)	3 Months. Participants of the drug management group will receive the drug interventions as azithromycin, remdesivir, favipiravir, infliximab, tocilizumab, siltuximab, hydrocortisone, rituximab, rintatolimod, intravenous immunoglobulin [15, 16]. The drug interventions will be directly prescribed by a physician specialized in treating PCC cases. A single brand name will be prescribed for each drug. We will communicate with the patients to ensure no adverse effects of the medications. The patient will be given a choice if they are willing to join the exercise programs; they have full liberty to join the programs even after the completion of the trial.
Intervention	Institute-based adapted physical activity and therapeutic exercise (APTE-I)	3 Months. APTE-I will be provided under the consultation of a consultant physiotherapist specializing in Post-COVID-19 Condition rehabilitation. Interventions will be provided for 45-minute sessions in a one-to-one approach. There might be some home exercises or advice to follow at home. There will be continuous communication with patients. To ensure that the treatment does not deteriorate the symptoms.

Inclusion Criteria

**Age From**	20.00 Year(s)
**Age To**	70.00 Year(s)
**Gender**	Both
**Details**	1) Diagnosis of long COVID according to who working group criteria1, 2) Diagnosis of chronic fatigue syndrome(CFS) according to the 2006 Canadian consensus criteria5, 3) Respondents meeting with persistent or relapsing chronic fatigue and post exertion malaise or fatigue criteria of the Canadian consensus guideline, and 4) willing to participate with the trail with a consent of adherence with the intervention

Exclusion Criteria

**Details**	(1) any preexisting post-exertion symptoms exaggeration, (2) any preexisting clinical condition with fatigue as cardiovascular or neurological disability, (3) any red flags or signs that are explained as contraindication according to safe Post-COVID-19 condition rehabilitation guideline, and (4) patient drop-out within the 1st week of inclusion

**Method of Generating Random Sequence**	Computer generated randomization
**Method of Concealment**	Sequentially numbered, sealed, opaque envelopes
**Blinding/Masking**	Participant, Investigator and Outcome Assessor Blinded

Primary Outcome

Outcome	TimePoints
Chalder fatigue Scale (CFS)	At baseline, 12 weeks and 6 months

Secondary Outcome

Outcome	TimePoints
Physical Functioning sub-domain of SF-36	At baseline, 12 weeks and 6 months
Disability Adjacent Life Years (DALYs)	At baseline, 12 weeks and 6 months

**Target Sample Size**	**Total Sample Size=**"124"<br>**Sample Size from India=**"124"<br>**Final Enrollment numbers achieved (Total)=** "Applicable only for Completed/Terminated trials"<br>**Final Enrollment numbers achieved (India)=**"Applicable only for Completed/Terminated trials"
**Phase of Trial**<br>Clarification(s) with Reply<br>Modification(s)	Phase 4
**Date of First Enrollment**	10/05/2024

**(India)** Clarification(s) with Reply Modification(s)	
**Date of Study Completion (India)**	Applicable only for Completed/Terminated trials
**Date of First Enrollment (Global)**	20/05/2024
**Date of Study Completion (Global)**	Applicable only for Completed/Terminated trials
**Estimated Duration of Trial**	**Years=**"0"<br>**Months=**"6"<br>**Days=**"0"
**Recruitment Status of Trial (Global)** Clarification(s) with Reply Modification(s)	Not Yet Recruiting
**Recruitment Status of Trial (India)**	Not Applicable
**Publication Details**	N/A
**Individual Participant Data (IPD) Sharing Statement**	**Will individual participant data (IPD) be shared publicly (including data dictionaries)?**<br><br>**Response** - YES<br><br>1. What data in particular will be shared?<br>**Response** - All of the individual participant data collected during the trial, after de-identification.<br><br>2. What additional supporting information will be shared?<br>**Response** - Study Protocol<br>**Response** - Statistical Analysis Plan<br>**Response** - Informed Consent Form<br>**Response** - Clinical Study Report<br><br>3. Who will be able to view these files?<br>**Response** - Anyone<br><br>4. For what types of analyses will this data be available?<br>**Response** - For individual participant data meta-analysis.<br><br>5. By what mechanism will data be made available?<br>**Response** - Proposals should be directed to [*altafhossainsarker272@gmail.com*].<br><br>6. For how long will this data be available *start date provided 31-05-2024 and end date provided 31-12-2028*?<br>**Response** - Immediately following publication. No end date.<br><br>7. Any URL or additional information regarding plan/policy for sharing IPD?<br>**Additional Information** - NIL
**Result Disclosure**	**Do you wish to upload results?**<br><br>**Response** - Summary results have not yet been disclosed
**Brief Summary**	ME/CFS patients will be recruited and treated in three specialized Hospitals. The study will be conducted in BRB Hospital Limited and Specialized Physiotherapy Hospital Ltd in the Dhaka division. In the Khulna division, the treatment center will be the Department of Physiotherapy and Rehabilitation at Jashore University of Science and Technology. The APTE-I, APTE-T, and DM groups will be recruited from any centers. However, the DM group will have respondents from the participants who are not willing to take any exercise interventions and only accept to take drug management. To prevent cross-contamination of the data,

	different treatments set up will be arranged in each study setting, and separate personnel will be employed for each treatment group.
