# Supplementary material for: Comparing effectiveness of physiotherapy versus drug management on fatigue, physical functioning, and episodic disability for myalgic encephalomyelitis in post-COVID-19 condition: a study protocol of randomized control trial
Source: Trials. 2024 May 15;25:321. doi: 10.1186/s13063-024-08077-x (PMC11094988; doi:10.1186/s13063-024-08077-x)
Supplement: Supplementary file 2 — Supplementary Material 2. [file 13063_2024_8077_MOESM2_ESM.docx]

**Consent Form**

Research Name: “Comparing Effectiveness of Physiotherapy versus Drug Management on Fatigue, Physical Functioning, and Episodic Disability for Myalgic Encephalomyelitis in Post-COVID-19 Condition (PCC)”.

Assalamu Alaiqum / Namaste,

I am Altaf Hossain Sarkar, Professor, Dhaka College of Physiotherapy, Affiliated under Dhaka University. I am currently doing Ph.D. research under the guidance of Professor Dr. Iqbal Kabir Jahid, Department of Microbiology, Jashore University of Science and Technology. The studies have Ethical ID numbers (BPA-IPRR/IRB/19/01/2023/69) and World Health Organization Primary Trial Registration numbers (CTRI/2024/01/061987). My research topic is " Comparing Effectiveness of Physiotherapy versus Drug Management on Fatigue, Physical Functioning, and Episodic Disability for Myalgic Encephalomyelitis in Post-COVID-19 Condition (PCC) "

This study is an experimental study and if you are interested to participate in this study, you will be asked a few questions. You can leave the questionnaire anytime during the question period. This data will be kept safe and will not be provided to anyone other than the patient's permission. It can take you 20 minutes to complete the entire question paper. Follow the instructions given in the questionnaire, if you need any help writing the answer you can take it. If you have something to know about this research, you can find out from me on the phone (01711-524435). Are you willing to participate in this research project voluntarily? Move forward if you have.

Identification number:

Participant's signature

Researcher's signature

Witness's signature
