# Supplementary figures and images for: Comparing effectiveness of physiotherapy versus drug management on fatigue, physical functioning, and episodic disability for myalgic encephalomyelitis in post-COVID-19 condition: a study protocol of randomized control trial
Source: Trials. 2024 May 15;25:321. doi: 10.1186/s13063-024-08077-x (PMC11094988; doi:10.1186/s13063-024-08077-x)

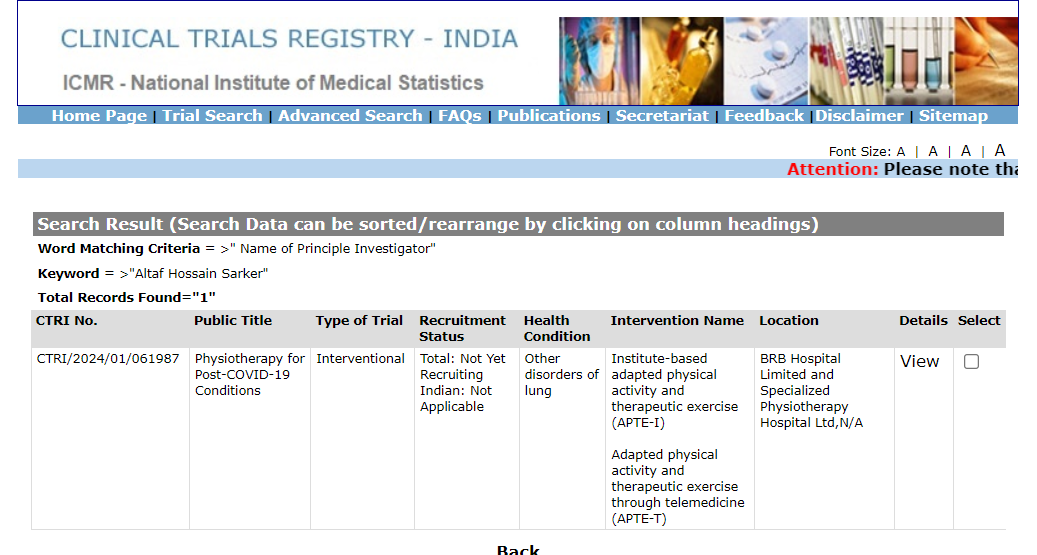

Supplement: Supplementary file 3 — Supplementary Material 3. [file 13063_2024_8077_MOESM3_ESM.png]
